# Exploring the relationship between anastasis and mitochondrial ROS-mediated ferroptosis in metastatic chemoresistant cancers: a call for investigation

**DOI:** 10.3389/fimmu.2024.1428920

**Published:** 2024-07-02

**Authors:** Yu Cao, Chang Lu, Narasimha M. Beeraka, Sergey Efetov, Mikhail Enikeev, Yu Fu, Xinyi Yang, Basappa Basappa, Mingze He, Zhi Li

**Affiliations:** ^1^ I.M. Sechenov First Moscow State Medical University of the Ministry of Health of the Russian Federation, Moscow, Russia; ^2^ Herman B. Wells Center for Pediatric Research, Department of Pediatrics, Indiana University School of Medicine, Indianapolis, IN, United States; ^3^ Raghavendra Institute of Pharmaceutical Education and Research (RIPER), Anantapuramu, Chiyyedu, Andhra Pradesh, India; ^4^ Laboratory of Chemical Biology, Department of Studies in Organic Chemistry, University of Mysore, Mysore, Karnataka, India

**Keywords:** anastasis, ferroptosis, chemoresistance, mitochondria, chemoresistance, EMT

## Abstract

Ferroptosis induces significant changes in mitochondrial morphology, including membrane condensation, volume reduction, cristae alteration, and outer membrane rupture, affecting mitochondrial function and cellular fate. Recent reports have described the intrinsic cellular iron metabolism and its intricate connection to ferroptosis, a significant kind of cell death characterized by iron dependence and oxidative stress regulation. Furthermore, updated molecular insights have elucidated the significance of mitochondria in ferroptosis and its implications in various cancers. In the context of cancer therapy, understanding the dual role of anastasis and ferroptosis in chemoresistance is crucial. Targeting the molecular pathways involved in anastasis may enhance the efficacy of ferroptosis inducers, providing a synergistic approach to overcome chemoresistance. Research into how DNA damage response (DDR) proteins, metabolic changes, and redox states interact during anastasis and ferroptosis can offer new insights into designing combinatorial therapeutic regimens against several cancers associated with stemness. These treatments could potentially inhibit anastasis while simultaneously inducing ferroptosis, thereby reducing the likelihood of cancer cells evading death and developing resistance to chemotherapy. The objective of this study is to explore the intricate interplay between anastasis, ferroptosis, EMT and chemoresistance, and immunotherapeutics to better understand their collective impact on cancer therapy outcomes. We searched public research databases including google scholar, PubMed, relemed, and the national library of medicine related to this topic. In this review, we discussed the interplay between the tricarboxylic acid cycle and glycolysis implicated in modulating ferroptosis, adding complexity to its regulatory mechanisms. Additionally, the regulatory role of reactive oxygen species (ROS) and the electron transport chain (ETC) in ferroptosis has garnered significant attention. Lipid metabolism, particularly involving GPX4 and System Xc- plays a significant role in both the progression of ferroptosis and cancer. There is a need to investigate the intricate interplay between anastasis, ferroptosis, and chemoresistance to better understand cancer therapy clinical outcomes. Integrating anastasis, and ferroptosis into strategies targeting chemoresistance and exploring its potential synergy with immunotherapy represent promising avenues for advancing chemoresistant cancer treatment. Understanding the intricate interplay among mitochondria, anastasis, ROS, and ferroptosis is vital in oncology, potentially revolutionizing personalized cancer treatment and drug development.

## 1 Introduction

Chemoresistance promotes cancer stemness by facilitating the release of exosomes containing peptides, nucleic acids, and various small molecules in multiple cancer types. This process converts drug-sensitive cells into cancer stem cells (1, 2). These cells represent a distinct subpopulation within tumor tissues, exhibiting self-renewal capabilities and giving rise to diverse tumor cell phenotypes (3). Chemoresistance could induce tumor heterogeneity (4–7), contributing significantly to tumor progression (8, 9). Chemoresistance can be modulated by the ferroptosis (9).

Cell death is a critical event within the physiological milieu and ferroptosis represents a significant kind of iron-dependent regulated cell death (RCD), distinct from established mechanisms such as apoptosis, pyroptosis, necrosis, and autophagy (10, 11). Ferroptosis is characterized by the generation of ROS and iron ion-dependent lipid peroxidation. Within cellular environments, ferroptosis is mediated by compounds like erastin or rat sarcoma virus oncogene homolog-selective lethal 3 (RSL3), which inhibit the expression of glutathione peroxidase 4 (GPX4) (12, 13). GPX4 is a selenoprotein that specifically catalyzes the conversion of lipid peroxide into lipids using glutathione. Consequently, reduced GPX4 levels disrupt the cellular oxidation balance. The Fenton reaction between ferrous ions and endogenous hydrogen peroxide generates hydroxyl radicals, which, in conjunction with polyunsaturated fatty acids, undergo multi-step free radical chain reactions, resulting in the formation of lipid peroxides (14). In a complete cellular system, ROS can stimulate mitochondria to open the permeability transition pore, amplifying the ROS signal through positive feedback. This positive feedback loop can be potentially regulated by mitochondria (15, 16). Consequently, when mitochondrial membrane integrity is compromised, the regulatory mechanism becomes impaired, leading to increased ROS production. After lipid peroxidation, modifications in the cell membrane is observed subsequently cause the generation of cytotoxic byproducts including malondialdehyde and 4-hydroxynonanaldehyde are generated, ultimately leading to the induction of ferroptotic cell death (17, 18).

When the term “anastasis” was introduced in 2012, it was associated solely with the reversal of apoptosis. This study explore novel cellular recovery phenomenon involving the reversal of ferroptosis in addition to anastasis, chemoresistance in cancer research.

Ferroptosis is a kind of programmed cell death reported in diseases including cancers (19, 20). Although the regulatory mechanisms and implications of ferroptosis continue to be elucidated, its potential resembles that of apoptosis in either augmenting cell death during cancer therapy or attenuating cell death to safeguard susceptible cells such as neurons, cardiomyocytes, and hepatocytes at the time of tissue damage (21).

**Figure 1:**
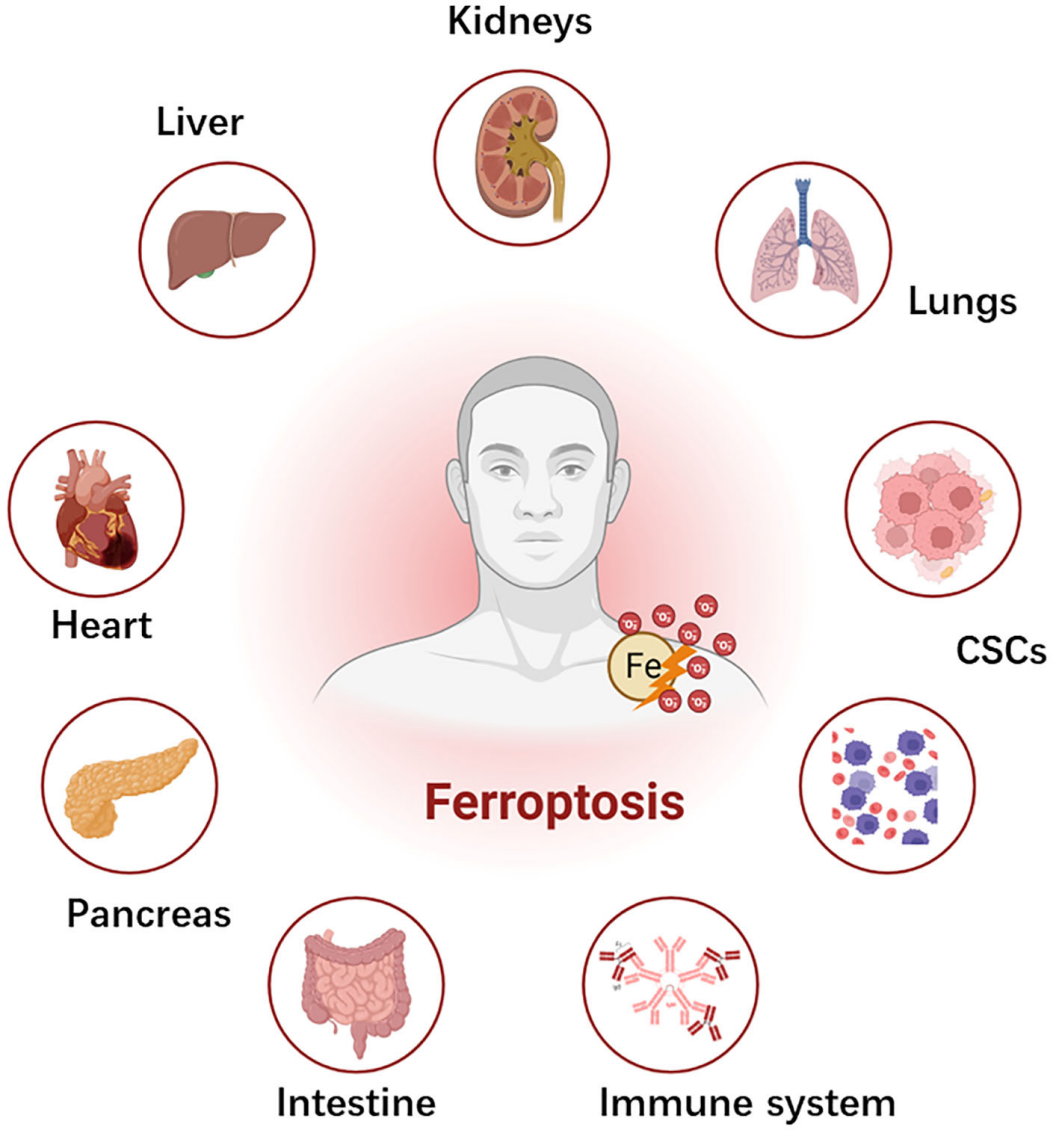
Schematic depiction of ferroptosis and systemic toxicities. Ferroptosis plays a pivotal role in several diseases across different organs. Ferroptosis has significant implications in the pathophysiology of chemoresistance and the development of cancer stem cells (CSCs), which yet require future studies in chemoresistant cancers (?). In the liver, it contributes to the development and progression of hepatocellular carcinoma, liver fibrosis, and ischemia-reperfusion injury. Within the cardiovascular system, it is implicated in ischemia reperfusion injury, transplantation, and atherosclerosis. Gastrointestinal system disorders, including gastric cancer and colorectal cancer, are influenced by ferroptosis. In the pancreas, it is associated with pancreatic cancer and type I diabetes mellitus. In the kidney, ferroptosis is involved in acute kidney injury, ischemia-reperfusion injury, and clear cell renal cell carcinoma. Lung diseases, such as lung cancer and acute lung injury, typically have ferroptosis-mediated pathophysiology. Hematological malignancies in the blood system and the suppression of T- cell immunological function in the immune system are also associated with ferroptosis.

For a significant duration, cell death was predominantly categorized into two main types: programmed cell death (PCD), exemplified by apoptosis mediated by cysteine aspartate-specific proteinase (caspase), and non-programmed cell death modalities such as necrosis (22–24) (**Table 1**). However, with advancements in molecular research within cell biology, an expanding repertoire of PCD modalities, including autophagy-dependent cell death, pyroptosis, and more recently, ferroptosis and cuproptosis, have been elucidated (47–49). Ferroptosis, a novel variant of programmed non-apoptotic cell death exerts a crucial regulatory influence on cancer progression. Growing evidence indicates ferroptosis induction as a promising avenue for cancer therapy. In recent years, several experimental reports described the role of mitochondria in various regulated cell death (RCD) processes, encompassing apoptosis, necrosis, pyroptosis, and ferroptosis (**Figure 1**). Thus, this study of ferroptosis reversibility offers novel insights for potential therapeutic interventions aimed at modulating cell death and survival through its reversible nature.

**Table 1:** History of classification and differences of cell death mode implicated in cell physiology.

S.No	Mode of Cell Death	Morphological characteristics	Core regulators	History	References
01	Necrosis	Cell volume expands, leading to organelle swelling and disintegration of the endoplasmic reticulum. Subsequently, the cells undergo self-dissolution, resulting in the release of cellular contents, often accompanied by inflammation in the surrounding tissues.	TNF, Toll, RIP, RIP1, MLKL, NPEPL1, COL4A3BP etc.	**Kerr et al., 1972** *shrinkage necrosis-* Observed in adrenal cortices of rats treated with prednisolone **Wyllie et al., 1973** *shrinkage necrosis- observed in* healthy neonatal rats **Crawford et al., 1972** Observed in developing vertebral arches of fetal rats.	(25–31)
02	Apoptosis	The cell membrane remains undamaged, with a decreased volume, leading to the formation of apoptotic bodies. There is no autolysis of the cells, no release of cellular contents, and no associated inflammatory response.	p53, Bax, Bcl-2, Caspases etc.	**Sydney Brenner, H. Robert Horvitz, and John E. Sulston -2002** *Nobel Prize* – for demonstrating the role of genes in modulating tissue and organ development and programmed cell death.	(32–35)
03	Autophagia	Formation of bilayer membrane structure with no changes of cell membrane.	ATG5, ATG7, Beclin 1, TOR, PI3K etc.	**Christian de Duve-1963** Gave the name autophagy **Yoshinori Ohsumi-2016** *Nobel Prize* – for demonstrating autophagy in yeast and the role of genes involved in autophagy	(36–40)
04	Ferroptosis	The cell membrane remains intact, but there is a decrease in mitochondrial volume characterized by densely packed mitochondrial membranes, a reduction or disappearance of mitochondrial cristae, and the occurrence of ruptures in the outer mitochondrial membrane.	GPX4, VDAC2/3, Ras, FANCD2, TFR1, p53, CISD1, SLC7A11, HSPB1 etc.	**Dolma et al., 2003** *Erastin*- Kill tumor cells with Ras gene mutation; Irreversible by caspase inhibitor. **Yang et al.,2008** *RSL3, RSL5*- Irreversible by iron-chelate agent DFOM and Vitamin E; ROS abnormality. **Dixon et al.,2012-** Ferroptosis official nomination- Caspase-independent; RPL8, IREB2, ATP5G3, CS, TTC35, ACSF-mediated mechanism; controlled by iron-chelate agent and antioxidant.	(19, 41–46)

Mitochondria play dual roles as key contributors to oxidative phosphorylation and primary generators of intracellular ROS (50, 51). Furthermore, they function as central hubs for various metabolic and signaling pathways, particularly fatty acid metabolism (52). Several studies have demonstrated that mitochondrial iron content, assessed through selective fluorescent iron indicators or electron paramagnetic resonance, constitutes a substantial portion, ranging from 20% to 50%, of the total cellular iron, depending on cell type (53–55). Mitochondrial iron plays a critical role in the formation of iron-sulfur clusters and heme synthesis. Notably, redox-active iron pools within mitochondria contribute to the accumulation of mitochondrial (MitoROS) (56–58). A distinctive feature of ferroptosis is its ability to evoke specific immune responses. During ferroptotic cell death, intracellular components are released into the extracellular milieu, triggering an immune reaction (59, 60). This response subsequently activates neighboring immune cells, such as macrophages, and facilitates the recruitment of additional immune cells to the site. It’s noteworthy that other forms of RCD may also induce immune responses, although the nature of the immune response varies depending on the type of RCD involved (61). Induction of ferroptosis in cells using erastin result in the reduced mitochondrial size compared to normal cells (62, 63).

In this review, we described a comprehensive exploration of the complex relationships and interactions between mitochondria, ROS, and ferroptosis-mediated chemoresistance and subsequently described the need for investigating the interplay of anastasis, EMT and mitochondrial ROS-mediated ferroptosis, and comparative efficacy of anticancer immunotherapeutics in metastatic chemoresistant cancers. This study is beneficial to several oncologists, molecular biologists, clinicians to develop personalized cancer treatment strategies to promote novel drug development.

### 1.1 Literature search

We undertook a significant literature analysis, sourcing data from different databases including Pubmed, Medline, eMedicine, Scopus, Google Scholar, the National Library of Medicine (NLM), and ReleMed. Our focus encompassed published reports and articles investigating the role of mitochondria, ROS, anastasis, ferroptosis-mediated chemoresistance, and immunotherapeutics in metastatic cancers.

## 2 Recent reports on intrinsic cellular iron metabolism & ferroptosis

Iron in dietary sources primarily exists as Fe (III) form, which undergoes reduction to Fe (II) within the intestinal lumen facilitated by brush-border membrane ferrireductase, duodenal cytochrome b (DCYTb), and other iron-reducing enzymes (64, 65). The resulting Fe (II) is then transported across the apical membrane of small intestinal epithelial cells (IECs) via divalent metal transporter 1 (DMT1/SLC11A2) and is subsequently sequestered within the labile iron pool (LIP) (66, 67). Intracellular Fe (II) is exported from IECs into the bloodstream via basolateral membrane ferroportin (FPN/SLC11A3), promoted through poly(rC)-binding proteins, and re-oxidized to Fe (III) by hephaestin (HEPH) (68, 69). Fe (III) is then bound to transferrin (TF) to form transferrin-bound iron (TBI) complexes, enabling circulation for transport to various tissues and organs. Within cells, TBI binds to transferrin receptor 1 (TfR1) and undergoes endocytosis, where Fe (III) is released into the cytoplasm, reduced back to Fe (II) by six-transmembrane epithelial antigen of prostate (STEAP3), and transported via DMT1/SLC11A2. A portion of cytoplasmic Fe (II) is stored in ferritin (FT) as the Fe^2+^-FT complex, while the remainder contributes to the LIP. Heme also plays a significant role in iron metabolism, entering cells through heme carrier protein 1 (HCP1) and feline leukemia virus subgroup C receptor 2 (FLVCR2), with subsequent release of Fe (II) catalyzed by heme oxygenase 1 (HO-1) for transport to the LIP (69–72). In addition, non-transferrin-bound irons (NTBI) in the cytoplasm of different tissues are transported to the cells through different NTBI transporters such as Zrt/Irt-like protein 8/14 (ZIP8/14, SLC39A8/SLC39A14), L-type and T-type calcium channels after which they are stored in the LIP (73–75). The process of detachment and release of Fe (II) from FT in Fe^2+^-FT is referred to as ferritinophagy, and it is regulated by nuclear receptor coactivator 4 (NCOA4). FT has heavy chain (FTH) and light chain (FTL) subunits. NCOA4 binds specifically to FTH1 and induces Fe^2+^-FT degradation, which separates FT from Fe (II) and thus allows Fe (II) release (76–80). A portion of intracellular Fe (II) enters the mitochondria through the MFRN1 and MFRN2 channels where they are then involved in the synthesis of heme and iron-sulfur cluster (ISC). ISC is an important inorganic cofactor that is widely engaged in a variety of biological functions including electron transfer which is the fundamental basis for cellular ATP production and protein synthesis. Meanwhile, recent studies have shown that ISC is tightly associated with DNA regulation (81–83).

**Figure 2:**
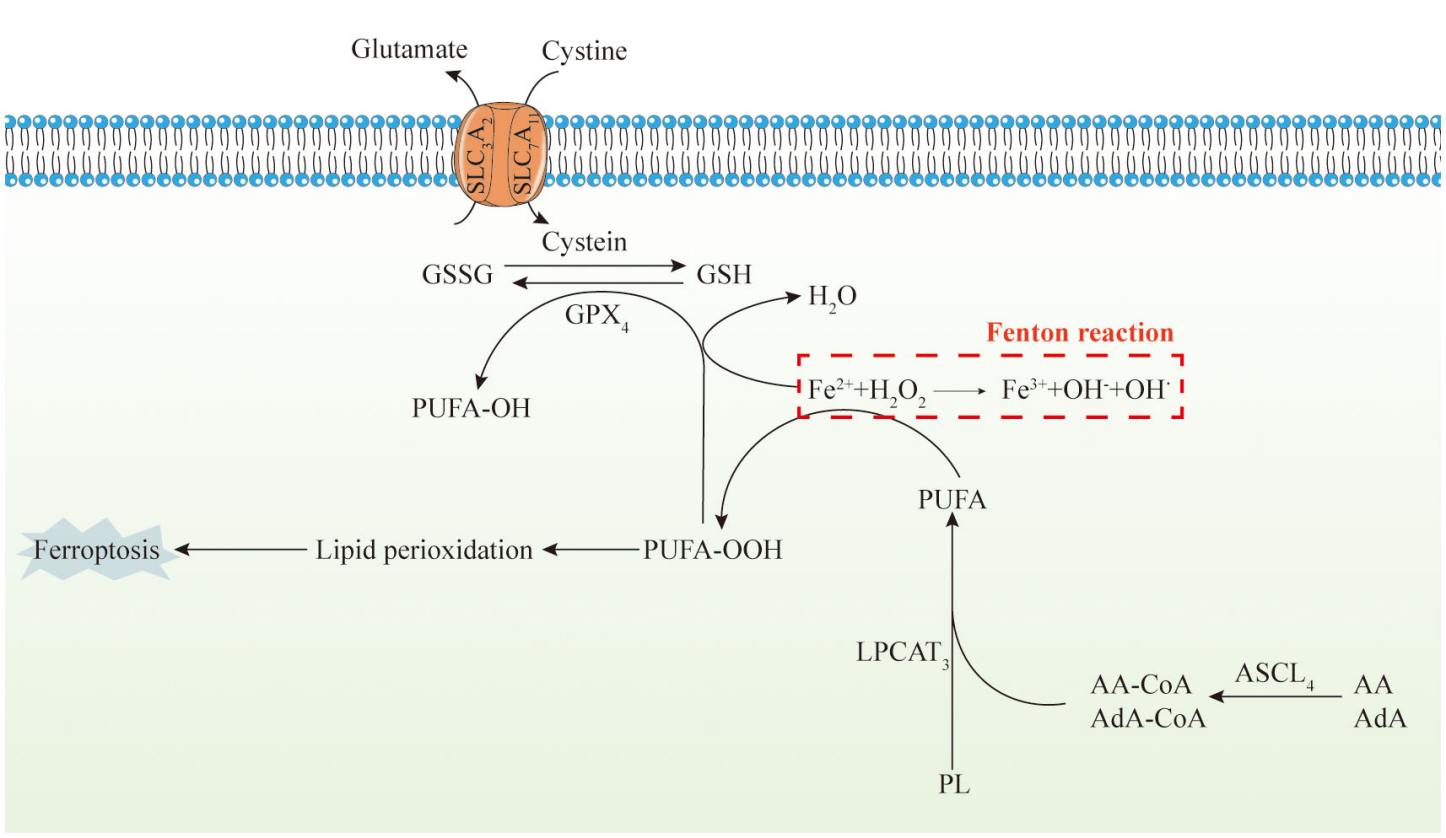
Mitochondria, iron metabolism, and ferroptosis. The diagram depicts the intricate series of events involved in iron metabolism within the human body, alongside ferroptosis, a form of regulated cell death. Initially, dietary iron, predominantly in the Fe(III) state, undergoes a sequence of conversions and transport processes. These processes include its reduction to Fe(II) within the intestinal environment, uptake by small intestinal epithelial cells, sequestration within the labile iron pool, extracellular release facilitated by ferroportin, and subsequent re-oxidation mediated by hephaestin. Subsequently, iron complexes with transferrin (TF), form transferrin-bound iron, which circulates in the bloodstream. TBI is internalized by cells via endocytosis, where it undergoes reduction to Fe(II) and enters the cellular cytoplasm. A fraction of the Fe(II) is stored within ferritin, while the remaining is reincorporated into the LIP. Heme, another significant iron source, follows a comparable pathway. Additionally, non-transferrin-bound iron from various tissues penetrates cells and is sequestered within the LIP. Furthermore, an additional reservoir of LIP is replenished through ferritinophagy, regulated by nuclear receptor coactivator 4. Intracellular free Fe(II) gains access to mitochondria via MFRN1 and undergoes subsequent biochemical transformations, ultimately leading to the onset of ferroptosis. Furthermore, ferroptosis is characterized by its dependence on iron and lipotoxicity. It operates by suppressing the activity of the lipid repair enzyme glutathione peroxidase 4 (GPX4), resulting in the accumulation of lipid hydroperoxides. Genetically, multiple genes modulate ferroptosis. Unlike other types of cell death characterized by extracellular manifestations, ferroptosis primarily unfolds intracellularly. This leads to distinctive cellular alterations, including reduced mitochondrial size, heightened membrane density, damaged cristae, fragmentation of the outer membrane without compromising the cell membrane, and minimal changes in nuclear morphology without chromatin condensation. Biochemically, the deficiency in peroxidation repair capacity primarily originates from the impairment of the phospholipid peroxidase GPX4. This deficiency triggers the acquisition of reactive iron and the oxidation of phospholipids containing polyunsaturated fatty acids (PUFA), ultimately inducing ferroptosis. The decrease in intracellular antioxidant capacity exacerbates lipid ROS accumulation, and cause cellular ferroptosis. Glutathione peroxidase is influenced by various pathways, including the XC−/GSH/GPX4 system, and the ACSL4/LPCAT3/15-LOX and FSP1/CoQ10/NAD(P)H pathways.

Mitochondrial Ferritin (FtMt) is a protein responsible for iron storage within cell mitochondria, sharing structural and functional similarities with cytoplasmic H-ferritin (84, 85). FtMt expression is confined to the mitochondria of the central nervous system and select oxygen-dependent tissues. Studies have indicated that erastin induces upregulation of Voltage-Dependent Anion Channel (VDAC) proteins on the mitochondrial membrane, thereby triggering ferroptosis (86–88). However, investigations on FtMt-SY5Y cells treated with erastin have shown unchanged levels of VDAC2 and VDAC3, suggesting a specific protective mechanism of FtMt against ferroptosis (89, 90). Upon erastin treatment, there is an upregulation of NADPH oxidase (NOX) proteins, critical for ROS generation in ferroptosis (91, 92). Interestingly, erastin-treated cells overexpressing FtMt exhibit a minimal increase in NOX2 levels, indicating inhibition of ROS generation by FtMt during ferroptosis (92, 93). Additionally, the mitochondrial glutaminolysis-tricarboxylic acid cycle-electron transport chain (TCA-ETC) axis plays a pivotal role in initiating ferroptosis. Within this pathway, ROS generation leads to ferroptosis through lipid peroxidation (94, 95) (**Figure 2**).

Dolma et al. (45) reported a compound within the erastin class that exhibited selective cytotoxicity against tumor cells specific for Ras gene mutations. This compound induced a distinct mode of cell death not previously documented, which was unaffected by caspase inhibitors. Subsequently, Yang et al. (46) identified two additional erastin analogs, RSL3 and RSL5, and demonstrated that changes in ROS levels could induce cell death. This process could be counteracted by iron chelators such as deferoxamine and vitamin E. Dixon et al. (10) formally coined the term “ferroptosis” to elucidate that this novel form of cell death relies on iron but is independent of the caspase activity. This study delineated the ferroptosis regulation by multiple genetic factors and elucidated its mechanistic association with the cystine/glutamate antiporter (System Xc-) (**Table 1**).

ROS encompasses a heterogeneous group of oxygen-containing reactive molecules, including superoxides, peroxide radicals, hydroxyl radicals, and alkoxyl radicals (96–98). Intracellular ROS are predominantly generated during ATP production within various cell types, representing normal metabolic byproducts of the mitochondrial respiratory chain. Furthermore, enzymes such as NADPH oxidase contribute to ROS production via enzymatic reactions (99, 100).

Polyunsaturated fatty acids (PUFA) constitute integral components of cell membrane phospholipid bilayers, crucial for maintaining membrane fluidity (101). Intracellular iron (II) (Fe (II)) participates in the Fenton reaction with hydrogen peroxide (H_2_O_2_), yielding hydroxyl radicals (OH•) that subsequently involved in lipid peroxidation reactions. Fe (II) itself catalyzes lipid peroxidation, promoting the formation of ROS such as PUFA-OOH, thereby causing cellular membrane damage (102–108).

The fundamental mechanism underlying ferroptosis involves the higher ROS generation, which leads to lipid peroxidation and disruption of cellular redox homeostasis, ultimately results in cell death that has significant implications in oncology. Despite the existence of various upstream regulatory pathways, it is widely acknowledged that the activity of glutathione peroxidase 4 (GPX4) directly or indirectly influences the onset of ferroptosis (13, 109–111).

## 3 Recent updated molecular reports on mitochondria’s role in ferroptosis and cancers

The principal morphological hallmark of ferroptosis is the changes in mitochondrial architecture and function, distinguishing it from other modes of cell death (112). Mitochondria undergoing ferroptosis display distinct alterations in morphology. Upon treatment with erastin, cells lack the characteristic morphological features typically associated with the absence of nuclear condensation, fragmentation, and necrotic lysis characteristic of necrosis; the lack of cellular crumpling, chromatin condensation, and apoptotic vesicle formation typical of apoptosis are changes associated with mitochondria-mediated ferroptosis. In response to erastin treatment, cells exhibit a distinct set of alterations characterized by a higher mitochondrial potential and membrane density. Furthermore, there is a reduction or disappearance of mitochondrial ridges, accompanied by the rupture of the mitochondrial outer membrane. These distinctive alterations serve as discernible criteria for distinguishing ferroptosis from apoptosis, necroptosis, and autophagy (113–118). These morphological alterations should be explored in various chemoresistant cancers.

### 3.1 The effect of tricarboxylic acid cycle and glycolysis on ferroptosis

The tricarboxylic acid (TCA) cycle and glutamine metabolism play integral roles in ferroptosis. While the precise metabolic pathways remain elusive, evidence suggests a close association between TCA cycle metabolites, enzymes, and ferroptotic cell death (119–121). In cellular contexts, blocking the cystine/glutamate antiporter (System Xc (-)) prevents the reverse transport of cystine and glutamic acid, thereby reducing cystine uptake, and causing intracellular ROS accumulation, lipid peroxidation, and initiation of ferroptosis (122). Elevated extracellular glutamate levels impede System Xc (-) function, consequently promoting ferroptosis.

Hence, diminishing glutamine (Gln) availability or inhibiting glutamine metabolism profoundly attenuates ferroptosis. Without adequate glutamine, mitochondrial damage and cell death induced by cystine starvation or erastin treatment are impeded (123, 124). Thus, glutamine is necessary for ferroptosis, and glutamine synthase (GLS) is a key regulatory factor for glutamine decomposition. GLS is divided into two isoforms: GLS1 and GLS2. GLS1 is mainly located in the cytoplasm, while GLS2 is located in the mitochondria (125–127). GLS1 and GLS2 catalyze the conversion of Gln to Glu (128, 129). Tal Hirschhorn et al. (130) found that ferroptosis can be prevented by drugs or gene inhibition of mitochondrial subtype GLS2. GLS2 has previously been shown to have activity consistent with tumor inhibition. GLS2 is a transcriptional target of p53 and upregulates during p53-dependent ferroptosis (131). Suzuki S et al. (132) found that GLS2-mediated glutamate can be converted into glutamic acid in the presence of aspartate aminotransferase (GOT) and glutamate dehydrogenase 1 (GLUD1). α - KG increases the production of ROS to downregulate the antioxidant defense function of cells, increase their sensitivity to iron-dependent cell death, and thus promote iron-dependent cell death. Downstream products of α-KG, such as succinic acid and fumaric acid, can also enhance cysteine depletion and cause cell ferroptosis (133). Several enzymes in the TCA cycle are necessary for iron-mediated ferroptosis caused by cystine starvation or erastin therapy, such as fumarate hydroxylase (FH), aconitase, and citrate synthesis (CS) (134–137). Therefore, reducing glutamine or blocking the glutamine decomposition typically impairs CDI ferroptosis. Without glutamine, cysteine starvation or erastin treatment cannot induce mitochondrial damage and subsequent cell death (123, 124).

Glycolysis serves as a crucial pathway for cells undergoing anaerobic respiration to generate energy. In tumor cells, metabolic rates are typically increased to sustain their rapid and uncontrolled proliferation. Metabolic shift is one of the significant physiological activities occurring in tumor cells to foster uncontrolled proliferation. Mainly, malignant cells rely on glycolysis to fulfill their energy demands while maintaining redox homeostasis to prevent ferroptosis (119, 138, 139). Ibtissam et al. (140) described that glycolytic cells exhibit significant inhibition of mitochondrial OXPHOS activity, mitigating ROS stress. However, this inhibition is reversible, and when glycolysis is suppressed, metabolic reconfiguration toward OXPHOS elevates cellular ROS levels, subsequently disrupting iron homeostasis and promoting lipid peroxidation, thus rendering tumor cells more sensitive to conventional chemotherapeutic agents and ferroptosis inducers. Hence, it is crucial to develop novel ferroptosis inducers to modulate the mitochondrial OXPHOS mainly to inhibit the development of CSCs in TME.

The altered function of VDACs may contribute to glycolytic alterations. Specifically, the opening of VDACs enhance mitochondrial substance exchange, subsequently increasing aerobic respiration efficiency (141, 142). VDACs, comprising VDAC1, VDAC2, and VDAC3 isoforms typically facilitate ATP, adenosine diphosphate, and mitochondrial-cytoplasmic exchanges (141, 142). Studies have indicated (143–145) that VDAC closure by microtubule proteins in a quiescent state restricts respiratory substrate influx into mitochondria, promoting glycolysis in cancer cells (Warburg effect), and this mechanism is yet to be studied in chemoresistant cancer cells. Yagoda et al. (146) revealed that cells activated by the RAS-RAF-MEK pathway can bind to certain VDAC ligands, subsequently enhancing susceptibility to erastin, a pivotal factor in cancer cell development. This heightened sensitivity prevents and reverses VDAC blockade by cytoplasmic free microtubule proteins, leading to VDAC opening and subsequent non-apoptotic cell death. Furthermore, intracellular free microtubulin abundance inhibits VDAC conductivity. N. DeHart et al. (86) described a novel mechanism of cell killing induced by erastin and erastin-like compounds, where the reversal of microtubulin-dependent VDAC inhibition fosters mitochondrial hyperpolarization and oxidative stress, culminating in mitochondrial dysfunction, dissipation of mitochondrial membrane potential (ΔΨm), and cell death. These mitochondrial mechanisms pertinent to the ferroptosis in chemoresistant tumor cells in other different cancer types should be explored typically by several preclinical studies.

### 3.2 Regulatory role of ROS and electron transport chain in ferroptosis

Mitochondria is the primary site for oxidative respiration within cells, where ETC activity not only facilitates ATP production but also plays a crucial role in generating ROS necessary for triggering cellular ferroptosis (147–149). For instance, glucose-dependent mitochondrial bioenergetic processes involve the transfer of electrons from complexes I and III in the ETC to molecular oxygen, resulting in ROS generation during catabolism (150–153). ROS, including hydrogen peroxide, superoxide anion, hydroxyl radicals, and peroxynitrite, are predominantly produced via mitochondrial metabolism and act as mediators of intracellular signaling pertinent to various forms of cell death including in tumor cells (16).

Electron leakage from ETC complexes I and III generates O2^·-^, which is consequently converted to H_2_O_2_ by disproportionation in the presence of superoxide dismutase. H_2_O_2_ reacts with Fe^2+^ to form hydroxyl radicals, initiating a chain reaction with PUFAs to induce cell death via the production of PUFA hydroperoxides (PUFA ⁃OOH) (154). Studies by Feng et al. (155) demonstrate that COX7A1, a cytochrome C subunit, inhibits mitochondrial autophagy and promotes susceptibility to ferroptosis in NSCLC cells by modulating the TCA cycle, ETC complex IV activity. This mechanism should be explored in several other cancer types.

Additionally, the inhibition of ETC mitochondrial complexes I, II, III, and IV attenuates ROS accumulation and ferroptosis induced by cysteine starvation or erastin et al. (134). Inhibition of mitochondrial complex III function with S3QEL significantly reduces lipid peroxidation and ferroptosis induced by cysteine starvation, highlighting the crucial role of superoxide produced by complex III in cysteine starvation-induced ferroptosis (156). The implications of cysteine depletion-induced ferroptosis in CSCs should be explored vividly by exploring the different cell signaling mechanisms.

Furthermore, ETC activity could influence the regulation of energy sensor adenosine 5’⁃monophosphate (AMP) and ROS levels. Mitochondrial electron transfer via the ETC complex generates a proton concentration gradient that powers ATP synthesis via ATP synthase. ATP production inhibits AMP-dependent protein kinase (AMPK) activity (157), promoting cellular ferroptosis by decreasing phosphorylation of acetyl-coenzyme A carboxylase (ACC) and increasing lipid synthesis, thereby influencing cellular susceptibility to ferroptosis (157).

### 3.3 Lipid metabolism and GPX4/system Xc^-^ in ferroptosis and cancers

**Figure 3:**
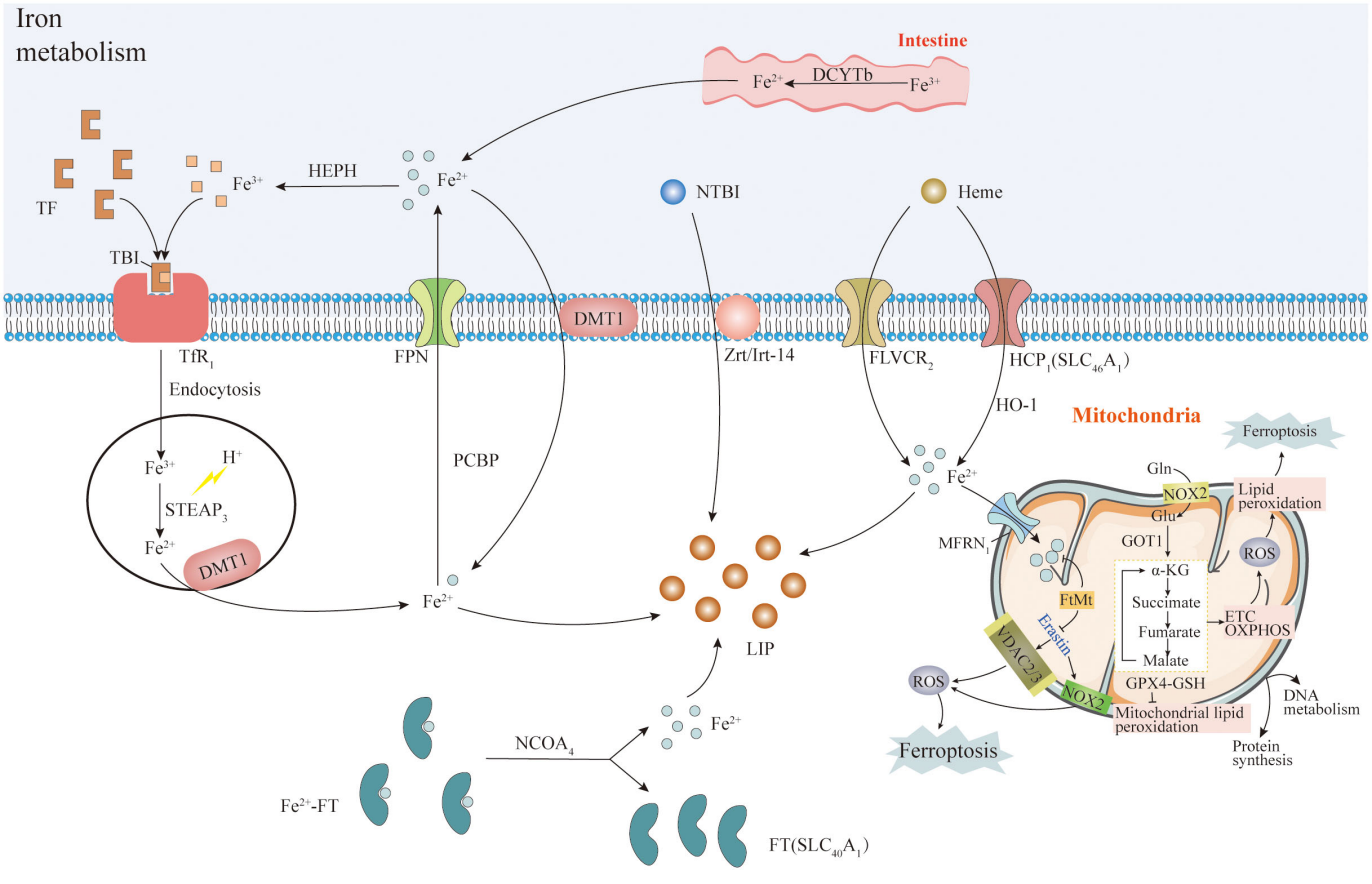
System Xc- and lipid metabolism. System Xc- is a transmembrane transporter composed of SLC3A2 and SLC7A11 (xCT) proteins. It transports intracellular glutamate and extracellular cystine, which is converted into cysteine. Cysteine plays a pivotal role in synthesizing the antioxidant glutathione. Glutathione exists mainly in reduced (GSH) and oxidized (GSSG) forms, maintained in balance by various mechanisms. GPX4, a selenoprotein, converts glutathione to GSSG and efficiently reduces phospholipid peroxides while decomposing H_2_O_2_. GPX4 also inhibits arachidonic acid activation and lipid peroxidation (ROS). Long-chain acyl-CoA synthetase 4 (ACSL4) activates arachidonic acid and adrenic acid into their CoA forms, promoting the activation of polyunsaturated fatty acids (PUFA) mediated by LPCAT3. Intracellular Fe(II) undergoes the Fenton reaction with H_2_O_2_, generating hydroxyl radicals (OH·), contributing to lipid peroxidation and ROS production, causing cell membrane damage. Note: The complete pathways explanation was given in subheading 3.

Lipids form the structural basis for cell membranes and organelles (mitochondria, endoplasmic reticulum), with lipid metabolism closely linked to cellular ferroptosis sensitivity via multiple pathways (47). System Xc- functions as a reverse cystine/glutamate transporter, comprising the transmembrane transporter proteins SLC3A2 and SLC7A11 (xCT). It transports intracellular glutamate and extracellular cystine, with intracellular cystine being converted to cysteine. Cysteine plays a crucial role in glutathione synthesis, primarily existing as reduced glutathione (GSH) and oxidized glutathione (GSSG), dynamically balanced within cells (158–161). GPX4, a selenoprotein, plays a pivotal role in maintaining this balance by catalyzing the conversion of glutathione to GSSG (122, 162). It effectively reduces phospholipid peroxides, decomposes H_2_O_2_ to water with GSSG, and inhibits arachidonic acid activation, thereby suppressing lipid peroxidation and ROS generation (22–24). As we discussed above, PUFAs are major substrates for lipid peroxidation during ferroptosis, damaging membrane structure and function. Enzymes involved in binding PUFA to phospholipids, such as acyl coenzyme A synthetase long-chain family member 4 (ACSL4), are integral to ferroptosis. ACSL4, besides being a sensitive indicator of iron-dependent cell death, regulates lipid metabolism by linking free fatty acids to CoA linkage, ultimately exchanged for phospholipids (163). ACSL4 specifically binds to substrates like arachidonic acid (AA) and adrenergic acid (ADA) (164) leads to the generation of fatty acyl CoA esters, esterified by lysophosphatidylcholine acyltransferase 3 (LPCAT3) into phospholipids like phosphatidyl ethanolamine (AA-ETA), predominantly found in the endoplasmic reticulum. Subsequent oxidation of AA-PE and ADA-PE by 15-lipoxygenase (ALOX15) produces lipid hydroperoxides, and other signaling molecules promoting ferroptosis (162, 165, 166). Recent studies highlight alternative pathways to ferroptosis, indicating that ACSL4/LPCAT3/15-LOX or p53/SLC7A11/12-LOX may cause lipid peroxidation during this process (167, 168). This underscores the complexity of ferroptosis regulation, offering potential therapeutic avenues for modulating cell death pathways to ameliorate chemoresistant-cancers (**Figure 3**).

## 4 Need for investigating the interplay between anastasis, ferroptosis, and chemoresistance and metastasis

Although both ferroptotic and apoptotic modes of cell death processes can be reversed, the underlying mechanisms facilitating these cell recovery phenomena remain to be fully elucidated in distinct cancer types. Primarily, both modes of cell death are typically different suggesting that different mechanisms may be required to halt the respective cell death mediators and repair the distinct types of damage for cell recovery. For instance, apoptosis involves the activation of proteases that can cause cleavage of distinct functional proteins, along with DNases like endonuclease G and DNA fragmentation factors, resulting in genomic destruction (169–171). Upon cessation of apoptotic stimulus, the cells that are undergoing recovery could induce upregulation of heat shock proteins and XIAP to impair stimulation of caspases, whereas the ICAD/DFF45 could cause inhibition of DNase activity, and PARP (a DNA repair enzymatic protein) to foster genome repair (172–174). In contrast, ferroptosis is characterized by the accumulation of ROS beyond the cell’s redox balance, which can cause lipid peroxidation followed by cell death (175). Administration of compounds like glutathione (GSH) or Fer-1 can facilitate ferroptosis reversal, potentially by enhancing GPX4 activity to mitigate ROS accumulation (176), while Fer-1 acts as a ROS scavenger to eliminate excessive cytosolic and lipid ROS, which could confer to the redox homeostasis and enabling cell recovery (177, 178).

Though glutathione (GSH) and Fer-1 are considered as inhibitors of ferroptosis, not all ferroptosis inhibitors possess the capacity to facilitate its reversal. For instance, compounds like aminooxyacetic acid (AOA), the iron chelator deferoxamine (DFO), dopamine, and vitamin C are known to inhibit ferroptosis initiation but do not promote cell recovery once ferroptosis has initiated. This limitation may stem from their targeting of upstream pathways involved in ferroptosis initiation, instead of conferring to the modulation of other downstream signaling pathways responsible for cell death. DFO functions as an iron chelator, depleting iron levels and hindering the iron-dependent accumulation of lipid ROS (19). Similarly, AOA inhibits transaminase activity, thereby blocking glutamine metabolism to alpha-ketoglutarate implicated predominantly involved in fatty acid synthesis (179). Dopamine prevents GPX4 degradation, while vitamin C scavenges free radicals in the aqueous phase (180–182). Previous studies (178) on the ferroptosis reversal through GSH and Fer-1 implicate regulators linked to GPX4 activity and lipid peroxidation as potential compounds that can induce ferroptosis reversibility. Unlike GSH and Fer-1, which act on mechanisms associated with removing lipid ROS, these inhibitors predominantly target the initiation of lipid peroxidation. This suggests potential differences in the regulation of preventing reversal of ferroptosis that warrant further investigation.

Several previous studies described the reversal of apoptosis when the removal of apoptotic stimuli is induced *in vitro* and *in vivo* studies (172–174, 178, 183–188). In contrast, another study (178) reveal that simply removing ferroptosis-inducing stimuli, including ‘erastin’ from HT-1080 cells or ‘erastin/glutamate’ from HT-22 cells, is insufficient to cause recovery of these ferroptosis-initiated cells. In this study (178), authors demonstrated the need to explore the possibility that certain types of dying cells with robust redox balance restoration capabilities may recover without the supplementation of GSH or Fer-1, a possibility that requires further investigation.

Ferroptosis can be reversed but raises fundamental inquiries that remain unanswered. For instance, can ferroptosis reversal occur within live animals? Tracking ferroptosis *in vivo* presents technical challenges, as cells recovered from ferroptosis appear morphologically identical to healthy non-ferroptotic cells. During the reversal of ferroptosis or apoptosis, cells encounter distinct forms of cellular damage. The existence of a common master regulator or signaling pathway triggering anastasis remains to be determined. Additionally, the broader physiological & therapeutic implications of this cellular recovery process require further exploration. Ongoing investigations aim to address these questions and identify the molecular regulators involved in these pathways. Understanding the signaling mechanisms and compounds that modulate ferroptosis and the reversal of ferroptosis could generate new approaches to investigate and comprehend the cell recovery process of anastasis (178).

Chemotherapy is a widely used treatment strategy aimed to ameliorate cancer cell proliferation (189, 190); however, some cells can survive chemotherapy, leading to cancer recurrence and metastasis (191–193). Even though various mechanisms, such as alterations in chemotherapeutic metabolism, changes in gene expression, metabolic reprogramming, stemness development, and changes in cell death pathways are significant influential factors which have been implicated in chemotherapy resistance (194–196), however, the precise underlying mechanisms remain unclear.

Apoptosis, a key mechanism targeted by chemotherapy, involves the activation of caspases, a group of cysteine proteases, ultimately leading to cell death. Previously, activation of executioner caspases was thought to be irreversible, marking a “point of no return” in apoptosis (197–199). Previous reports revealed anastasis process, wherein cells could refrain from apoptotic stress although executioner caspase activity is evident (174, 184, 200). Anastasis, or cellular survival following stress-induced activation of executioner caspases, has been documented in a subset of mammalian cell lines following exposure to various chemical stressors and chemotherapeutic agents (172, 173, 184, 201–204).

Studies on different cancer cell types, including breast (108, 205–208), melanoma (117), cervical, and ovarian cancers (209), have shown that anastasis confers new traits upon cancer cells, such as increased drug resistance and migration (172, 173, 201–204, 210). For instance, anastatic breast and cervical cancer cells are associated with enhanced chemoresistance and migration capabilities (203). Similarly, melanoma cells surviving executioner caspase activation caused through the upregulation in the expression of transient tBid or exposure to the dacarbazine (a chemotherapeutic drug) demonstrate elevated cell migration *in vitro* and increased metastasis *in vivo*.

According to previous reports (202), a lineage tracing system was employed to identify and isolate cells undergoing executioner caspase activation and their progeny. These studies suggest that anastatic colorectal cancer cells exhibit a higher metastasis, and chemoresistance due to increased upregulation in the expression of cIAP2 and NF-κB activity. According to this study, NF-κB activation and cIAP2 expression conferred by chemotherapeutics are crucial for anastasis (202). Exposure to chemotherapeutic drugs triggers NF-κB activation and upregulation of cIAP2 expression in colorectal cancer cells, promoting anastasis. Therefore, activated NF-κB and cIAP2 establish a positive feedback loop within anastatic cells, further enhancing migration and metastasis (202).

Thus, anastasis cause colorectal cancer cells with heightened migratory potential. Similar observations of higher metastasis following survival from stress-induced executioner caspase activation were evident in various tumor types, including cervical (173), breast (203), melanoma (202), and ovarian cancers (204), suggesting that a higher migration may represent a typical phenotypic alteration associated with anastasis. Furthermore, apoptosis and executioner caspases are typically involved in mediating the metastasis of lymph nodes (211–213). However, anastatic cells maintained enhanced migration independently of apoptotic cells, suggesting that executioner caspase activity is dispensable for sustaining increased migratory capacity in anastasis (214, 215). This observation aligns with previous reports on melanoma cells (202).

Various cancer cells exhibit increased migration following anastasis, with diverse underlying molecular mechanisms. Inhibition of TGF-β signaling moderately mitigated anastasis-induced migration in HeLa cells following brief ethanol exposure (173). Another report showed that the blocking of nuclear export reversed heightened migration in anastatic breast cancer cells (203). Similarly, a previous study (202) concluded that melanoma cells surviving executioner caspase activation resulted in augmented motility through the JNK hyperactivation.

Ru Wang et al., 2013 (216) revealed that cIAP2 enhances migration in anastatic colorectal cancer cells in an NF-κB-dependent manner, emphasizing role of cIAP2 as a positive regulator of migration. According to previous reports, NF-κB has a significant role in proliferating cancer cells and promotes migration or metastasis by modulating the downstream signaling cascades including STAT3 and MMPs (217–219). cIAP2 has previously been associated with drug resistance in pancreatic cancer, colorectal cancer (220, 221), and oral squamous cell carcinoma (222, 223), further supporting its role in mediating chemoresistance. A positive feedback loop between cIAP2 and NF-κB fostered levels of cIAP2 expression and NF-κB activity in anastatic cells, enhancing their migratory potential. Thus, cIAP2/NF-κB signaling and anastasis are critical regulators of post-chemotherapy metastasis (216). Additionally, Ru Wang et al., 2013 revealed (216) demonstrated a cIAP2-dependent increase in chemoresistance in anastatic colorectal cancer cells.

Studies on *Drosophila notum* epithelium demonstrated that (224) the ability of stressed cells to undergo anastasis may be relied upon executioner caspase activation. By inhibiting caspase-3 and 7, cIAP2 can foster a higher executioner caspase activity (225, 226), thereby promoting anastasis. Molecular mechanisms governing the anastasis regulation through cIAP2, NF-κB, and regulators which were already investigated in previous studies warrant further elucidation in future research for several other chemoresistant cancers.

## 5 Integrating ferroptosis with chemoresistance

Drug-resistant cancer cells, along with neighboring stromal cells, may create a protective environment, allowing vulnerable cancer cells to evade ferroptosis and chemotherapy. For example, cancer-associated fibroblasts (CAFs) could foster a reduction in cisplatin accumulation, subsequently conferring resistance to both chemotherapy and ferroptosis (21). Ferroptosis is intricately controlled by a multifaceted network involving epigenetic, pre-transcriptional, post-transcriptional, and post-translational modifications (227). Tumor cells, characterized by heightened sensitivity to signals regulating cell survival and death, consequently cause increased susceptibility to ferroptosis. Various factors that promote tumor growth, the buildup of iron, enhanced lipid synthesis, or undergoing epithelial-to-mesenchymal transition (EMT), can augment susceptibility of tumor cells to ferroptosis. Additionally, numerous chemotherapeutic agents elicit anti-cancer efficacy through the induction of oxidative stress, disrupting the anabolism of genetic components thereby synergizing with ferroptosis to ameliorate cancers. Nonetheless, the precise targeting of tumor cells by promoting ferroptosis induction remains a challenging task in current oncological strategies (9). Tumor drug resistance encompasses several intricate mechanisms, with disruption of redox homeostasis emerging as a pivotal contributor. Tumor cells exhibit greater resistance to oxidative stress by modulating ROS generation, thereby acquiring drug resistance (228). Ferroptosis typically linked with mitochondrial oxidative stress and ROS production, plays a significant role. Alterations in oxidative stress regulation can modulate ferroptosis, consequently influencing tumor cell sensitivity to chemotherapeutics. This association depends upon a nuanced balance between lipid peroxidation and the accumulation of ROS promote ferroptosis, thereby suppressing tumor proliferation; as we discussed the reduction of lipid peroxidation and ROS levels facilitates tumor cell survival, promoting resistance to anti-tumor agents. The dynamic interplay between ROS generation and scavenging mechanisms lies at the core of this relationship, which require future studies pertinent to several chemoresistant cancers (9).

As we discussed previously, chemotherapy remains a significant therapeutic modality treating numerous solid malignancies, but the challenge of chemoresistance significantly limits its effectiveness (229). Interestingly, a significant link has been observed between resistance to cisplatin and resistance to ferroptosis within tumor environments. Malignant cells resistant to cisplatin often exhibit increased expression of genes associated with ferroptosis resistance (230–232). For instance, cisplatin-resistant cancer cells overexpress the Wnt pathway membrane receptor, frizzled homolog 7 (FZD7), leading to activation of TP63 and subsequently enhanced GPX4 expression (233, 234). Various cancer types develop cisplatin resistance by upregulating system Xc− expression and maintaining high levels of GSH (235–237). Recent studies indicate that cisplatin treatment could induce ferroptosis in tumor cells, suggesting the significant efficacy of chemotherapy in triggering ferroptosis (238, 239).

CAFs have been implicated in reducing cisplatin accumulation, thereby promoting resistance to both chemotherapy and ferroptosis by supplying glutathione (GSH) and cysteine to ovarian cancer cells (240). Another study described that upregulation of miR-4443 in NSCLC cells, and undergoes exosomal transfer to sensitive cells, and enables upregulation of FSP1 expression. This elevated FSP1 expression is crucial to evade ferroptosis. Conversely, CD8+ T cells induce the generation of IFN-γ subsequently reduces system Xc− activity in cancer cells, thus fostering reduction in cisplatin resistance through modulation of the JAK/STAT pathway (240).

The significant interplay between chemoresistance and the underlying ferroptosis mechanisms is yet to be explored vividly to develop novel therapeutic modalities against ferroptosis-resistant tumor cells (239). Combinatorial administration of GPX4 inhibitors and system Xc− inhibitors & cisplatin has been shown to overcome chemotherapy resistance, enhancing the anti-cancer response in various tumors (238, 241, 242). However, these mechanisms require future studies in several other chemoresistant cancer types.

**Figure 4:**
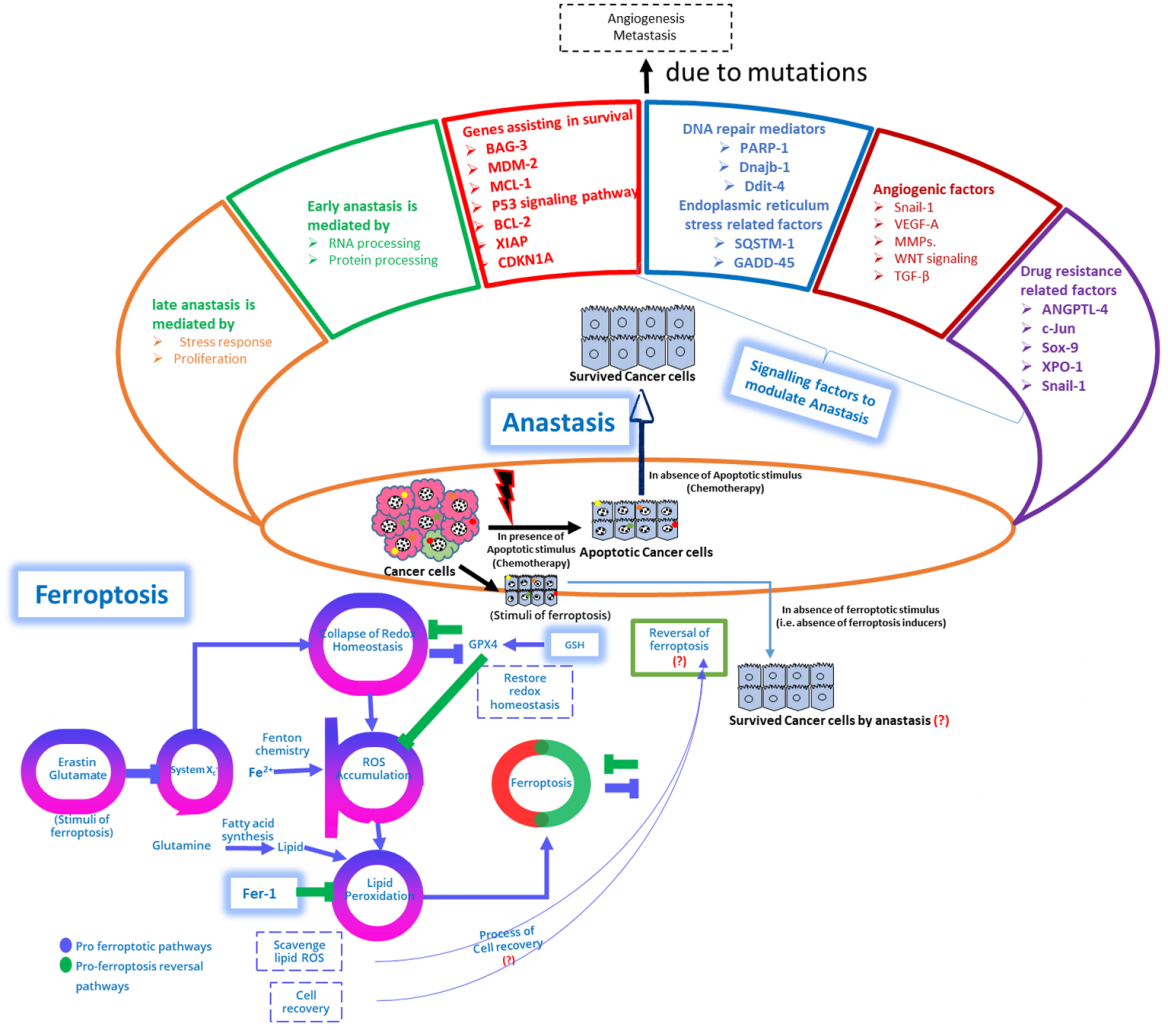
Schematic depiction of genomic variations within genes regulating apoptosis are evident in cancer and contribute to the activation of pro-proliferation and pro-survival pathways, leading to apoptosis resistance. Upper Panel: While apoptosis has traditionally been regarded as irreversible, emerging evidence suggests its potential reversibility, termed anastasis, wherein cells can escape apoptotic signals even after reaching advanced stages. Anastasis poses challenges for chemotherapy development and utilization. This figure demonstrates the multifaceted role of survival mechanisms, metastasis, epithelial-mesenchymal transition (EMT), and DNA damage repair in facilitating anastasis, and the mechanistic interplay between ferroptosis reversal and anastasis to modulate chemoresistance, (?) emphasizing its implications for drug resistance and therapeutic strategies. Lower Panel: A proposed model illustrates the reversal of ferroptosis, and the interplay between pro-ferroptosis reversal pathways and pro-ferroptosis pathways is discussed. The schematic representation depicts structural and molecular changes during anastasis, showing cancer cells recovering from apoptosis by activating anastasis and acquiring mesenchymal features and molecular alterations promoting survival, metastasis, and angiogenesis. Genes associated with anastasis-mediated drug resistance, including *ATF3, c-FOS, c-JUN, INBHA, SNAIL-1, ANGPTL4*, and *SOX-9*, are highlighted. Cells recovering from apoptosis through anastasis exhibit pronounced structural changes, alter focal adhesion kinases, activate genes related to the actin cytoskeleton, and elevate proteins associated with chemoresistance. In addition, the interplay between the anastasis, and mitochondrial-ROS modulated ferroptosis proposed mechanisms should be explored to demonstrate the underlying mechanisms pertinent to chemoresistance with anastasis, and ferroptosis reversal (178, 250).

Chemoresistance specific to the anti-glioma agent, temozolomide (TMZ) is typically linked to resistance to ferroptosis induced by the overexpression of system Xc− and GSH (243, 244). Moreover, cancer cells can alter the iron homeostasis by lipocalin-2, subsequently causing ferroptosis resistance to 5-fluorouracil (245). The phenotypes of chemoresistant tumors closely mimic resistance to ferroptosis, particularly focusing on crucial targets such as GPX4, system Xc−, FSP1, and NRF2 (6, 246–249). Hence, it is crucial to profile these ferroptosis targets in chemoresistant tumors (**Figure 4**).

Yet, the adaptive reaction to chemotherapeutics includes the activation of MTDH gene, promoting the mesenchymal phenotype of tumors and augmenting chemoresistance. However, diminishing the expression of GPX4 and system Xc− can increase susceptibility to ferroptosis. Thus it has been demonstrated that the ferroptosis resistance emerges as a new rationale underlying tumor recurrence following chemotherapy (21).

The surgical intervention combined with adjuvant multi-drug chemotherapy is another strategy of therapeutic modality for patients diagnosed with pancreatic ductal adenocarcinoma to enhance survival rates. However, the persistence of gemcitabine resistance in PDAC cells, continues to challenge clinicians (251). Recent insights suggest a correlation between gemcitabine resistance (252, 253) and ferroptosis resistance, primarily driven by the hyperexpression of system Xc−. Notably, inhibiting system Xc− in PDAC cells leads to glutathione (GSH) depletion *in vitro*, triggering ferroptosis and restoring sensitivity to both gemcitabine and cisplatin (254).

Changes in apoptosis-regulating genes are common in cancer, promoting activation of pro-proliferation and pro-survival pathways, leading to resistance against apoptosis. Contrary to apoptosis irreversibility, emerging evidence suggests its potential reversibility, termed anastasis, enabling cells to evade apoptotic signals even at advanced stages, challenging chemotherapy development (178, 250). It is crucial to explore the multifaceted role of survival mechanisms, metastasis, EMT, and DNA damage repair in facilitating anastasis. It also explores the interplay between ferroptosis reversal and anastasis in modulating chemoresistance, highlighting implications for drug resistance and therapeutic strategies. Future studies are warranted to examine ferroptosis reversal, and the interaction between pro-ferroptosis reversal and pro-ferroptosis pathways. The schematic representation **Figure 4** delineates structural and molecular changes during anastasis, with cancer cells activating anastasis to recover from apoptosis, acquiring mesenchymal features, and molecular alterations promoting survival, metastasis, and angiogenesis. Genes linked to anastasis-mediated drug resistance, such as ATF3, c-FOS, c-JUN, INBHA, SNAIL-1, ANGPTL4, and SOX-9, are emphasized (178, 250). Cells recovering from apoptosis via anastasis undergo notable structural changes, altering focal adhesion kinases, activating genes associated with the actin cytoskeleton, and increasing chemoresistance-associated proteins. Additionally, exploring the interplay between anastasis and mitochondrial-ROS modulated ferroptosis mechanisms can elucidate underlying mechanisms of chemoresistance with anastasis and ferroptosis reversal (178, 250)

Intricate interplay pertinent to the anastasis, chemoresistance, and ferroptosis resistance in tumors require studies related to therapeutic efficacy of several therapeutic strategies. The recurrence of tumors following chemotherapy may, in part, be attributed to ferroptosis resistance. This underscores the significant chemotherapeutic efficacy of combinatorial regimens. In this context, targeting the tumor microenvironment (TME) and metabolic pathways to enhance ferroptosis sensitivity in malignant cells could emerge as a significant therapeutic strategy for overcoming the challenges posed by chemoresistance (21).

Emerging evidence indicates that ferroptosis can mitigate acquired resistance to several targeted therapies, including lapatinib, erlotinib, trametinib, dabrafenib, and vemurafenib (255–257). Notably, tumor cell lines resistant to these targeted drugs often exhibit markers of EMT and display heightened susceptibility to ferroptosis (255–257). EMT, characterized by the loss of epithelial characteristics and acquisition of mesenchymal properties, is known to confer chemoresistance in tumor cells, driven by transcription factors such as TWIST1 and ZEB1. For instance, β-elemene has been shown to sensitize KRAS mutant colorectal cancer cells to cetuximab through a ferroptotic mode of cell death while blocking EMT (257). Thus, ferroptosis could modulate chemoresistance to the chemotherapeutics and appear intricately linked to EMT processes.

Additionally, agents targeting PD-1 and PD-L1 garnered approval from the FDA for treating various malignancies. Anti-PD-L1 antibodies have been observed to foster lipid peroxide-mediated ferroptosis in proliferating cancer cells, with the efficacy of anti-PD-L1 antibody therapy being compromised by ferroptosis inhibitors (258). Combining anti-PD-L1 antibodies with ferroptosis inducers has shown significant efficacy in inhibiting tumor growth, attributed to the release of interferon-γ by cytotoxic T cells, which activates STAT1 and inhibits xCT expression, thereby promoting ferroptosis (258). Implicating ferroptosis inducers that stimulate STAT1 activation may overcome tumor resistance to immunotherapy, providing a wide range of potential for the clinical implementation of ferroptosis inducers in combination with immune checkpoint inhibitors (ICIs) (9).

## 6 Integrating anastasis with EMT and chemoresistance and immunotherapeutics

Cancer stem cells (CSCs) pose significant challenges for developing new therapies due to their strong association with tumor relapse and chemoresistance (259, 260). Cells that are undergoing anastasis exhibit resistance to various apoptotic phases. Previous reports in cancer metastasis demonstrated that cells recovering from apoptotic phase show a higher CD44 expression, a key marker that signifies chemoresistance, and these cells exhibit properties similar to CSCs. Additionally, the apoptosis reversal induces epigenetic modifications in the gene promoters related to CD44 and CD24 genes (201), further contributing to their stem-like characteristics. It is crucial to examine functional role of anastatic mechanisms for the formation and maintenance of CSCs by the prospective studies. Anastasis harnesses conventional prosurvival and pro-metastatic factors to impair the apoptosis process. Actuation of EMT and the related EMT modulators is considered as crucial aspect to modulate cell survival and for maintaining stemness (261, 262). For instance, among these modulators, SOX-9, a gene involved in tumor growth, angiogenesis, differentiation, and survival, plays a critical role. SOX-9 is also linked to chemoresistance against gemcitabine, underscoring its significance in chemoresistance and the need for exploring its role in anastasis subsequently to identify targeted therapeutic strategies to overcome these challenges (263, 264).

Understanding the molecular signatures of anastasis through preclinical or clinical studies is a significant aspect in cancer research (172). Anastasis was observed in mouse models where primary murine liver cells and NIH3T3 mouse embryonic fibroblast cells were exposed to ethanol to induce apoptosis. Following the treatment, cells were subjected to washing and allowed to recovery phase in apoptosis by exposing them into a fresh media (172, 184). Transcriptomic analysis from the cells obtained from recovery phase of this apoptosis revealed a significant upregulation of genes linked to survival pathways; these genes are BCL-2 family members (BAG-3, BCL-2, and MCL-1), XIAP, MDM2. In addition, the genes related to heat shock proteins including Dnajb1, Hsp90aa1, Hspa1b, and Hspb1 were found to be upregulated in this recovery phase (172, 184) (**Figure 4**). Notably, residual apoptotic markers dissipated during early phase of anastasis initiation. Inhibiting key survival factors such as Hsp90, MDM2, XIAP, and BCL-2 significantly impaired anastasis, highlighting the significant dependency on these cellular survival mechanisms (172, 184).

Cancer cell survival is not solely dependent on anti-apoptotic gene expression. It also relies on genes involved in regulating cell cycle, and DNA repair; In addition, the survival of these proliferating cancer cells relies on gene expression pertinent to angiogenesis, migration, and TGF-β modulation, and these genes are reported to be upregulated at the time of early anastasis phase (172). Previous reports on HeLa cells recovering from ethanol-induced apoptosis revealed significant increases in c-Fos, c-Jun, Klf4, and Snail-1 expression (**Figure 4**). These findings imply that the upregulated genes at the recovery phase and post-apoptosis induction are primarily linked to proliferation and pro-survival signaling (173). On the contrary, genes activated during late anastasis predominantly affect actin cytoskeleton rearrangement, and ribosome biogenesis (**Figure 4**) (173, 200). At the time of anastasis, the involvement of several survival factors includes MDM2 revealing that the p53 signaling implications include CDKs, expression of E2F and cell cycle factors (265). Other investigations into the AP-1 transcription factor actuation, which includes c-Jun and c-Fos; these genes are significantly involved in mediating pro-apoptotic and pro-survival factor expression. Hence, their actuation during anastasis offers insights into the balance between pro-survival and pro-apoptotic signaling which is yet to be explored vividly (266–268).

Many proteins that play roles in promoting cell proliferation and preventing apoptosis are also critical for recovering cells from death, which indicates that cancer cells can trigger survival mechanisms under stress conditions, resulting in significant molecular transformations that restore the cell to its normal state (173, 183). During anastasis, structural changes such as actuation of focal adhesion kinases and reorganization of the actin cytoskeleton (173, 183). Due to these changes, recovered cancer cells undergo migration. Furthermore, Snail-1 expression is significantly increased during the initial anastasis phase. When Snail-1 is silenced using shRNA, the recovery process, including migration, is impaired, leading to increased PARP-1 cleavage and sustained activation of cell death pathways (173, 183). Early phase of cell recovery involves the coordinated actuation of TGF-β signaling, which is crucial for the survival of the cells followed by migration. Additionally, MMP-9, MMP-10, MMP-13, VEGF-A, and Angpt-l4 are other EMT and angiogenic factors that could play a significant functional role in promoting the cell recovery from cell death (173, 183).

Furthermore, EMT plays a crucial role in modulating the process of metastasis, anastasis, and stemness, making it a promising target for combinatorial drug design (81) (250, 269). Salinomycin, mocetinostat, and metformin could effectively target EMT and they were studied significantly for their potential to overcome chemoresistance when used alongside drugs like doxorubicin, 5-FU, and gemcitabine (270–276). Trichostatin A and vorinostat are HDAC inhibitors that can inhibit EMT by downregulating E-cadherin expression and modulating the expression of HIF-1α and NF-κB (277–280). In cancers with EGFR mutations, diindolylmethane (DIM) and its analogs have demonstrated potential in preventing EMT and metastasis, suggesting their suitability for use in combinatorial regimens along with DNA-damaging agents (281, 282). Combinatorial regimen of DIM with camptothecin can prevent drug-induced EMT in the murine models associated with Apc-floxed colorectal cancers (283). Recurrent administration of therapies such as chemo/radio-therapies can cause side effects by co-activating DNA repair followed by cell survival and EMT pathways, which are crucial in anastasis. For instance, the nuclear translocation of nucleases pertinent to apoptosis such as AIF and Endo-G are evident at the time of anastasis when observed during reversal of ethanol-mediated DNA damage (283).

Critical involvement of DNA damage response (DDR) proteins during anastasis yet require future studies for several other cancers. DDR proteins, such as ATM, ATR, and DNA-PK, are known to play pivotal roles in detecting and repairing DNA damage, thereby maintaining genomic stability. Their activation during anastasis suggests a potential mechanism by which cells recover from near-death states and restore proliferation (184). Given the established link between robust DNA repair mechanisms and poor cancer prognosis (284), it is imperative to further investigate how these pathways contribute to anastatic processes and chemoresistance. Elucidating these mechanisms may reveal new therapeutic targets to improve cancer treatment outcomes, particularly in tumors that exhibit high levels of DNA repair activity. Studies should focus on understanding the specific DDR pathways (284) involved in anastasis and their impact on the efficacy of chemotherapeutic agents, potentially leading to the development of novel strategies to overcome resistance.

Cancer cells exert chemoresistance when administered recurrent cytotoxic chemotherapeutics to several cancer types, and EMT plays a pivotal role in mitigating drug susceptibility (270, 285–287). At the time of treating cancer cells with DNA-damaging agents, specifically epithelial-origin cancer cells start expressing mesenchymal markers. As previously discussed, EMT is closely associated with survival factors, in addition, the drug-induced EMT leads to a significant activation of survival factors such as NF-κB, c-FLIP, survivin, as well as AKT. Notably, markers pertinent to early apoptosis were found to occur alongside EMT and cell survival proteins. Moreover, EMT modulator vimentin was shown to hinder process of apoptosis (283). EMT is significantly higher in drug-induced metastasis than in spontaneous metastasis. For instance, autochthonous breast cancer murine model was studied where transgenic mice with a Cre recombinase construct driven by the Fsp-1 promoter (288) which would express Cre recombinase in mesenchymal lineage cells undergoing EMT. This system was designed so that when the Fsp-1 promoter was actuated, the Cre recombinase could induce knock out of RFP in another transgenic construct that included GFP (288). Consequently, cells expressing GFP indicated EMT activation. According to this study, spontaneous lung metastasis had few GFP-positive cells, whereas cyclophosphamide-treated mice had significant GFP expression in metastatic lung nodules (288). GFP-positive cells confined to the regions of metastasis exhibited a higher chemoresistance than the GFP-negative cells (288). These findings highlight the complexity of drug-induced EMT and *in vivo* actuation of anastasis.

As previously discussed, NF-κB plays a critical role in promoting cell survival, making it a key target in emerging cancer therapies. Bortezomib, for instance, inhibits NF-κB by preventing the degradation of IκB, thereby suppressing NF-κB signaling pathways. This therapeutic approach in combinatorial regimen with temozolomide and paclitaxel resulted in promising results (289). Furthermore, several NF-κB inhibitors such as curcumin, BMS-345541, and bindarit exhibit significant efficacy (290). These compounds could be particularly effective when used as combinatorial regimen with DNA-damaging drug molecules to counteract anastasis, a process through which cells survive and recover from apoptosis phases. This combination strategy aims to enhance the efficacy of cancer treatments by targeting the molecular mechanisms involved in evading apoptosis and chemoresistance. Further research and clinical trials are essential to validate these findings and optimize combination therapies for better clinical outcomes.

## 7 Immunotherapy treatment with ferroptosis and chemoresistance

Previous studies reported the combinatorial regimen of immunotherapeutics along with novel ferroptosis modulators against cancers. Anti-cancer immunity is higher with GPX4 inhibitors against triple-negative breast cancers (TNBCs) specifically LAR subtype. The synergistic effect of GPX4 inhibition and PD-1 blockade demonstrates a higher efficacy compared to individual therapeutic interventions *in vivo* settings (291). In glioma, wherein ferroptosis serves as the predominant mechanism of programmed cell death (PCD), it fosters an immunosuppressive tumor microenvironment facilitated by tumor-associated macrophages, thereby promoting malignant progression and unfavorable clinical outcomes. Targeting ferroptosis inhibition is suggested as a promising strategy to augment the immune checkpoint blockade (ICB) efficacy (292).

In melanoma, a predictive model termed the Ferroptosis Score (FPS) was developed utilizing 32 ferroptosis-related genes to prognosticate cancer outcomes. Elevated FPS levels are indicative of a more dynamic tumor immune microenvironment and enhanced responsiveness to immune checkpoint blockade (ICB), such as PD-1 blockade (293). BEBT-908, an inhibitor targeting PI3K and HDAC, induces immunogenic ferroptotic cell death in cancer cells, augmenting ICB therapy efficacy by upregulating MHC class I expression and activating IFN-γ signaling (294). Statins facilitate immunotherapy by inducing ferroptosis in non-small cell lung cancer cells while inhibiting PD-L1 expression (295). Inhibition of Ferroptosis Suppressor Protein 1 (FSP1) significantly induces ferroptosis in cancer cells and promotes immune cell infiltration, including dendritic cells, macrophages, and T cells. Combinatorial therapy with immunotherapy and FSP1 inhibition typically mitigates hepatocellular carcinoma burden *in vivo* (296). Inflammation-associated ferroptosis biomarkers positively correlate with PD-L1 expression, high microsatellite instability, tumor mutational burden, and ICB response rates (297). Short-term methionine starvation synergistically promotes ferroptosis with CD8+ T cells by stimulating CHAC1 transcription, enhancing ICB therapy. Combined treatment involving intermittent methionine starvation, system xc- inhibition and PD-1 blockade exhibits potent anti-tumor effects (298). Ferritin Light Chain (FTL) promotes cancer cell ferroptosis in glioblastoma, polarizing tumor-associated macrophages towards the M2-type, thus fostering a pro-tumor TME. Inhibiting FTL represents a promising strategy to sensitize glioblastoma to PD-1 blockade (299). However, further investigations are warranted to evaluate the efficacy of these ICBs in combination with ferroptosis modulators against chemoresistant cancers (21).

## 8 Conclusions and future prospects

Recent advancements in cancer research and development have significantly shifted the focus from merely identifying anti-cancer compounds to unraveling the intricate molecular mechanisms underpinning cell survival and drug resistance. Current studies described the molecular signaling pathways between tumors and their microenvironment, examining how these interactions affect cellular survival and resistance to therapy-induced apoptosis. This review highlights various cellular mechanisms that enable cancer cells to evade apoptosis and acquire oncogenic phenotypes. Two pivotal concepts emerging from this research are mitochondria-modulated ferroptosis in cancer cells, anastasis, which introduces a novel perspective on cancer-related therapies by fostering novel investigations on chemotherapeutics or immunotherapeutics to mitigate chemoresistant cancers.

As previously highlighted, ferroptosis is characterized by iron-dependent toxicity, excessive ROS accumulation, and consequent lipid peroxidation, disrupting cellular redox balance and culminating in membrane damage, ultimately triggering cell death (10, 102, 300). Excessive oxidative stress induces irreversible damage to mitochondrial function and integrity, leading to energy depletion and ferroptosis (88). Ferroptosis manifests distinct morphological, genetic, and biochemical features when compared to apoptosis, necrosis, and autophagy (12, 108, 301). Numerous studies have implicated ferroptosis in diverse biological processes and pathogeneses, prominently in cardiovascular diseases, brain lesions, renal impairment, and various malignancies, where it plays a pivotal regulatory role within the tumor microenvironment (63, 302–307).

Additionally, cancer cells exhibit a higher demand for iron metabolism compared to normal cells, rendering them more susceptible to ferroptosis induction (305, 308). Research further suggests a close nexus between ferroptosis and the inhibition of tumor cell growth. Activation of ferroptosis can impede cancer cell proliferation, thereby influencing the efficacy of tumor immunotherapy and patient prognosis (309–313). Consequently, targeting or inducing ferroptosis in cancer cells is emerging as a novel therapeutic strategy (314). This comprehensive review elucidated the intricate interplay between ferroptosis, mitochondria, and iron metabolism, underscoring the potential translational impact of these findings in oncology.

In prostate cancer (PCa), it has been proposed that the ferroptosis initiators erastin and RSL3 could profoundly inhibit PCa cell progression without adverse events (315–317). In pancreatic cancer, gemcitabine is the primary treatment, albeit with unsatisfactory efficacy (167). Heat shock protein family A5 (HSPA5) has been closely linked to the prognosis of gemcitabine-treated patients (318). Zhu et al. (318) suggested that HSPA5-GPX4 pathway modulation to contribute to chemoresistance in pancreatic cancer cells against gemcitabine, and blocking of HSPA5 or GPX4 gene expression potentially reversing this resistance. Ferroptosis has also been implicated in this process.

In hepatocellular carcinoma (HCC), sorafenib remains the principal chemotherapeutic drug. Sun et al. (319) found that sorafenib could induce HCC cell death by increasing oxidative stress. However, the ferroptosis inhibitor ferrostatin-1 could counteract this process, indicating the significance of ferroptosis in the mechanism of action of sorafenib. Furthermore, a combination of erastin, sorafenib, and haloperidol could induce a higher intracellular iron concentration, inducing the Fenton reaction and excessive ROS production, ultimately leading to HCC cell ferroptosis (320).

In TNBC, Ding et al. (321) demonstrated that the inhibitor DMOCPTL ubiquitinated GPX4 through EGR1 regulation, inducing TNBC cell ferroptosis. The fundamental mechanism of action and clinical value of ferroptosis remain incompletely understood. Indeed, potential valid ferroptosis-related biomarkers warrant further investigation. Moreover, it remains unclear in clinical treatment whether inducing ferroptosis in tumor cells would impair liver and kidney functions in patients. Developing ferroptosis-inducing agents capable of selectively eliminating chemoresistant tumor cells while sparing normal cells remains an area requiring further exploration. Basic research and clinical translation of ferroptosis encounters myriad uncertainties and obstacles. As foundational research on ferroptosis progresses, the clinical application of ferroptosis inducers and inhibitors may become viable. Thus, research endeavors focused on ferroptosis hold considerable potential to confer substantial benefits to cancer patients with chemoresistance in the future by exploring the interplay between ferroptosis, anastasis, and chemoresistance. Specifically, can the reversal of ferroptosis manifest *in vivo* within live animals? Tracking ferroptosis *in vivo* poses technical challenges, given the morphological similarity between cells recovered from ferroptosis and healthy non-ferroptotic cells. Additionally, while cells undergoing reversal of ferroptosis or apoptosis must address distinct forms of cellular damage, it is plausible that they may share a common master regulator or signaling molecule initiating the process of anastasis. Moreover, elucidating the long-term physiological, pathological, and therapeutic aspects of anastasis modulators remains imperative (178).

The fundamental mechanism underlying ferroptosis involves the interplay of iron metabolism and lipid peroxides. The identification of FSP1 as a negative regulator expands our understanding of ferroptosis. Extensive investigations into ferroptosis have unveiled numerous potential therapeutic targets for combating chemoresistant tumors. These discoveries hold promise for the development of novel drugs and treatment modalities targeting these specific pathways. Presently, resistance to conventional chemotherapy, targeted therapy, or immunotherapy agents represents a major challenge in the management of refractory tumors or recurrent malignancies. Given the ubiquitous nature of chemoresistance mechanisms, tumors that exhibit resistance to one drug often display varying degrees of resistance to others within the same class, with some even demonstrating multidrug resistance, significantly compromising treatment efficacy and patient survival. Multiple studies have demonstrated that ferroptosis can enhance tumor cell death synergistically with anti-tumor drugs, thereby augmenting drug efficacy and potentially reversing drug resistance, offering a promising therapeutic avenue for patients with drug-resistant tumors. Notably, tumor cells exhibit heightened sensitivity to iron overload and ROS accumulation compared to normal cells, rendering targeting of iron ions a feasible approach to enhance treatment efficacy and mitigate side effects.

Integration of cross-disciplinary technologies, such as nano-vehicle agents (322) combined with ferroptosis, may represent a more tailored approach to tumor treatment. However, several challenges must be addressed before clinical application. Firstly, while numerous *in vitro* methods exist for assessing ferroptosis and anastasis, the development of sensitive and accurate *in vivo* evaluation techniques remains elusive. Secondly, the specificity and safety profile of compounds targeting ferroptosis regulators must be significantly evaluated in preclinical and clinical settings to minimize adverse effects. Lastly, further elucidation is warranted regarding which tumor types or characteristics, such as specific gene mutations, are particularly susceptible to ferroptosis. Nonetheless, comprehensive investigations into the molecular signaling underlying ferroptosis, anastasis, and tumor drug resistance hold considerable significance to revolutionize tumor diagnosis and treatment, offering new avenues for therapeutic intervention (9). Continued research is warranted to develop personalized combinatorial therapeutic regimens against cancers to modulate these molecular signaling that optimize therapeutic efficacy and overall survival.

## Author contributions

YC: Methodology, Investigation, Formal analysis, Data curation, Conceptualization, Writing – review & editing, Writing – original draft. CL: Writing – original draft, Formal analysis, Data curation. NB: Writing – original draft, Validation, Project administration, Data curation, Writing – review & editing, Supervision, Methodology, Investigation, Conceptualization. SE: Writing – review & editing, Project administration, Methodology, Investigation. ME: Writing – review & editing, Methodology, Investigation, Formal analysis. YF: Writing – original draft, Project administration, Methodology. XY: Writing – original draft, Software, Resources. BB: Writing – review & editing, Validation, Project administration. MH: Visualization, Resources, Project administration, Funding acquisition, Writing – review & editing, Supervision, Methodology. ZL: Validation, Software, Resources, Project administration, Methodology, Data curation, Conceptualization, Writing – review & editing, Writing – original draft, Visualization, Supervision, Investigation, Funding acquisition, Formal analysis.
